# Identification of Rice Sheath Blight through Spectral Responses Using Hyperspectral Images

**DOI:** 10.3390/s20216243

**Published:** 2020-11-02

**Authors:** Fenfang Lin, Sen Guo, Changwei Tan, Xingen Zhou, Dongyan Zhang

**Affiliations:** 1 School of Remote Sensing & Geomatics Engineering, Nanjing University of Information Science & Technology, Nanjing 210044, China; 002173@nuist.edu.cn; 2 National Engineering Research Center for Agro-Ecological Big Data Analysis & Application, Anhui University, Hefei 230601, China; P19301119@stu.ahu.edu.cn; 3 Jiangsu Key Laboratory of Crop Genetics and Physiology, Yangzhou University, Yangzhou 225000, China; cwtan@yzu.edu.cn; 4 Plant Pathology Lab, Texas A&M AgriLife Research Center, 1509 Aggie Drive, Beaumont, TX 77713, USA; xzhou@aesrg.tamu.edu

**Keywords:** rice sheath blight, spectral response, “three-edge” parameters, hyperspectral imaging, narrow-band vegetation index, remote sensing

## Abstract

Sheath blight (ShB), caused by *Rhizoctonia solani* AG1-I, is one of the most important diseases in rice worldwide. The symptoms of ShB primarily develop on leaf sheaths and leaf blades. Hyperspectral remote sensing technology has the potential of rapid, efficient and accurate detection and monitoring of the occurrence and development of rice ShB and other crop diseases. This study evaluated the spectral responses of leaf blade fractions with different development stages of ShB symptoms to construct the spectral feature library of rice ShB based on “three-edge” parameters and narrow-band vegetation indices to identify the disease on the leaves. The spectral curves of leaf blade lesions have significant changes in the blue edge, green peak, yellow edge, red valley, red edge and near-infrared regions. The variables of the normalized index between green peak amplitude and red valley amplitude (Rg − Ro)/(Rg + Ro), the normalized index between the yellow edge area and blue edge area (SDy − SDb)/(SDy + SDb), the ratio index of green peak amplitude and red valley amplitude (Rg/Ro) and the nitrogen reflectance index (NRI) had high relevance to the disease. At the leaf scale, the importance weights of all attributes decreased with the effect of non-infected areas in a leaf by the ReliefF algorithm, with Rg/Ro being the indicator having the highest importance weight. Estimation rate of 95.5% was achieved in the decision tree classifier with the parameter of Rg/Ro. In addition, it was found that the variety degree of absorptive valley, reflection peak and reflecting steep slope was different in the blue edge, green and red edge regions, although there were similar spectral curve shapes between leaf sheath lesions and leaf blade lesions. The significant difference characteristic was the ratio index of the red edge area and green peak area (SDr/SDg) between them. These results can provide the basis for the development of a specific sensor or sensors system for detecting the ShB disease in rice.

## 1. Introduction

Sheath blight (ShB), caused by *Rhizoctonia solani* AG1-IA, is one of the three major diseases in rice. The disease can cause yield losses of 10% to 30%, with most severe loss of up to 50% [1,2,3,4]. The successful control of ShB is crucial to the profitable of rice production [5]. Accurate detection of rice ShB is the key to develop effective management strategies for control of this disease. Remote sensing technology can be a useful tool to quickly, efficiently and accurately detect and monitor the occurrence and development of ShB and other crop diseases and insect pests [6,7,8].

Studies have been conducted using RGB, multispectral and hyperspectral images to detect ShB in rice. Researchers often use visual technology and neural network methods to extract color and texture features of ShB at different levels of disease, and investigate the feasibility of image processing technology in the detection of ShB in rice. However, the results of these studies are easily affected by background, light, growth period, etc. [9,10,11]. Multispectral sensors contain more wavebands such as near-infrared wavelengths, which are used for calculation of some broad-band vegetation indices. Qin et al. [12] made an early investigation to examine the applicability of multispectral remote sensing to detect rice ShB. In this case, it was discovered that the multispectral sensors with four broader bands were not able to accurately discriminate plants with light infections. In addition, Zhang et al. [13] also used RGB and multispectral sensors to assess disease severities of rice ShB, and showed that the normalized difference vegetation index (NDVI) could better distinguish the disease degree than other indices but the accuracy was not high.

Hyperspectral technology has been proved to be a useful tool to detect crop diseases. The occurrence of diseases may cause modifications to the biophysical and biochemical characteristics of plant tissue, which results in changes in reflectance of leaf tissue. Hyperspectral remote sensing has advantages of high spectral resolution with many wavebands. Specific wavebands can be selected for the detection of diseases with different symptoms [7,14,15]. Most application strategies have been on the establishment of identification models combined with hyperspectral data reduction methods [16,17,18,19]. These models contain partial least squares-discriminant analysis, linear discriminant analysis, a back propagation neural network or a support vector machine. Principal component analysis and the minimum noise fraction transformation method are adapted as data reduction methods. However, spectral response characteristics of plant leaves or canopy infected with ShB are not clearly analyzed in this process, and these discriminant models are largely dependent on specific data.

The development of classification models is useful and important in advancing portable tools for rapid assessment of detecting plant diseases. However, the prerequisite for achieving a model with high accuracy is the construction of specific and optimal indicators for the disease of interest. These indexes can be reflectance of a waveband or some bands, spectral characteristic position variables, or some vegetation indices sensitive to the disease. This depends on the detailed exploration of spectral response mechanism for the disease. The progression and spread of rice ShB can be divided into three types, namely, “inward spread”, “vertical spread” and “horizontal spread” [3]. When rice is infected by the ShB fungus, lesions with fuzzy edges initially appear on the leaf sheath near the surface water line. Then, runner hyphae infect and spread vertically upwards along leaf sheaths and leaf blades, and horizontally from leave to leave and from plant to plant [1]. Plant leaves or canopies with ShB are the main field of view for remote sensing. In this study, we used the ground-based imaging spectrometer to obtain the hyperspectral remote sensing images of rice leaves naturally infected with ShB. According to disease symptoms, hyperspectral data of rice leaves infected with ShB were carefully processed to explore the spectrum response mechanism of rice ShB. 

The objectives of this study were to analyze variations of the reflectance spectrum curves of rice leaf blades with different levels of ShB. According to spectrum change rules, “three-edge” parameters and vegetation indices closely related to rice ShB were selected to construct the spectral characteristic library of rice ShB. Then, we examined the applicability of these spectral features to identify rice ShB at the leaf scale. The spectral differences between leaf sheath lesions and leaf blade lesions were also discussed in order to establish a basis for multi-angle remote sensing monitoring of ShB in rice.

## 2. Materials and Methods

### 2.1. Field Sampling

**Figure 1:**
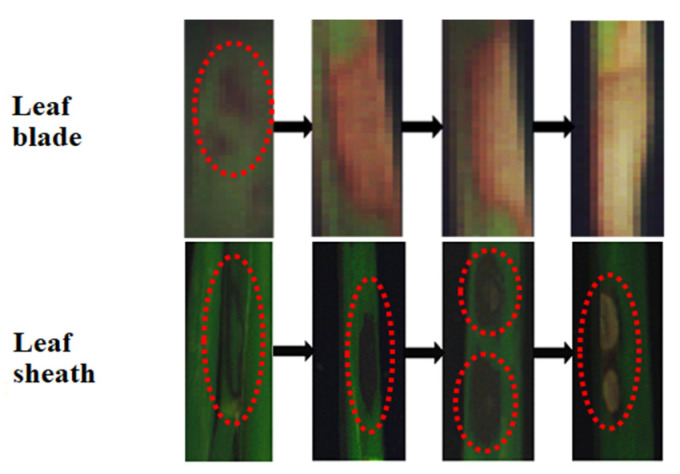
Symptom development of sheath blight (ShB) on the rice leaf blade and the leaf sheath.

Diseased samples were collected from a rice field trial conducted in Gongdao Town, Yangzhou, Jiangsu Province (32°34′37″ N, 119°19′23″ E) in July through August 2019. Rice variety used in this study was “Nanjing 9108”. Diseased samples were collected at both the jointing and booting stages of rice growth. Samples collected at the jointing stage were leaf sheaths with the symptoms of ShB. Samples collected at the booting stage were leaf blades (fully expanded leaves) since at this stage, the disease had spread from the lower to upper portion of the canopy. Figure 1 shows the progress of the ShB symptoms from mild to severe on the rice leaf blade and the leaf sheath. On the leaf blade, small dark green patches appeared at the early stage of the disease development. With the development of the lesion, the lesion expanded, turning brown in color. At a certain stage, the center of the lesion became dry and was light in color, but its surrounding area was still brown. Finally, the lesion turned white and completely dried up, leading to the death of the leaf. On the leaf sheath, dark green, water-stained lesions appeared at the early stage of infection, and gradually changed into elliptic or even large gray-white or gray-green clouded lesions, from outer to inner sheaths with a bleached center and an irregular purple brown border. Furthermore, the ShB symptoms at different development stages simultaneously appeared in various areas of the leaf blade and the leaf sheath. 

To collect desirable samples, entire rice plants with root system were pulled out, placed in ice boxes, and transported to the laboratory in the same day for processing. The roots were cut off and the plant samples were wiped clean and placed in a dark room for scanning.

### 2.2. Hyperspectral Image Acquisition

Hyperspectral images were acquired in a dark room using the visible–near-infrared hyperspectral imaging system that consists of three components. These components are the imaging part, the light source, and a mobile platform. The imager was the main part and was composed of an imaging spectrograph (ImSpector Pro-V10E; Spectral Imaging Ltd., Oulu, Finland), a charge-coupled device (CCD) camera and a camera lens (HSIA-OL25). Spectral resolution of the imaging spectrograph was 2.73 nm. The light source consisted of four tungsten halogen lamps placed on both sides of the camera at a 45° angle, with a light controller. The mobile platform was a conveyer belt driven by a stepping motor. The whole system was controlled by a computer with SpectraView software. Scanning of rice samples was completed by moving the conveyer belt while the imager was fixed. A white panel was used as the reference every time the rice samples were scanned under the same illumination conditions.

Hyperspectral images were obtained on 31 July and 22 August. Three data sets were extracted from these images. The first data set consisted of 136 images with different development stages of leaf blade lesions, the second data set consisted of 44 images with different levels of ShB severity on the whole leaves, and the third data set consisted of 58 images with different development stages of leaf sheath lesions.

### 2.3. Calibration of Hyperspectral Images

The acquisition of imaging spectral data under the indoor conditions can be affected by many factors, including instrument parameters and light source. The flat field (FF) model was used to reduce noise and convert the spectral radiance measurements into reflectance. The FF model is developed on the basis of the internal average relative reflectance (IARR) model. There is a region with a certain area and uniform distribution in an image before using this method. The average value of all pixels in the uniform region was calculated, and then the value of each pixel in the image was divided into the average value. The formula is as follows:

(1) $$R_{j}\left(i\right)=\left(\frac{N_{j}^{a}}{\sum_{i}DN_{j}\left(i^{a}\right)}\right)DN_{j}\left(i\right)$$

where *R_j_*(*i*) is the spectral reflectance of the *i*th pixel in the jth channel of the sensor; *N^a^_j_* is the total number of pixels of the *j*th channel in the uniform region; *DN_j_*(*i^a^*) is the digital number (DN) value of the *i*th pixel in the *j*th channel in the uniform region; and *DN_j_*(*i*) is the DN value of the *i*th pixel in the *j*th channel in an image. Here, the white reference panel is treated as the uniform region.

### 2.4. “Three-Edge” Parameters and Narrow-Band Vegetation Indices

In order to fully explore spectral features of ShB lesions on the leaf blade and the leaf sheath, two spectral analysis methods, “three-edge” parameters and narrow-band vegetation indexes [20,21,22], were used in this study.

Diseases can cause changes in physiological and chemical substances and cellular structures in plants, resulting in spectral changes in the regions of the blue edge, green peak, yellow edge, red valley, red edge and near-infrared. The blue edge, green peak, yellow edge, red valley, red edge and near-infrared band are in the range of 490–530 nm, 510–560 nm, 550–582 nm, 640–680 nm, 670–750 nm and 783–890 nm, respectively. An increase in the amount of chlorophyll, for example, causes a red-edge shift towards longer wavelengths. A series of spectral characteristic parameters are constructed in these ranges, including position, amplitude, area, ratio and normalized ratio [20,23,24,25]. These are termed “three-edge” parameters. A total of 36 representative “three-edge” parameters are included in Appendix A, with abbreviations and definitions of each variable.

High spectral resolution amplifies spectral information. Narrow-band vegetation indices are designed for hyperspectral data. Some indices closely related to diseases have been proved to be useful to detect the changes in plant parameters, such as pigment content, nitrogen content, water content, and leaf area or biomass. The selected vegetation indexes are shown in Appendix B [26,27,28,29,30,31,32,33,34,35]. 

### 2.5. The Method of Estimating the Relevance of Attributes to Rice ShB

Relief algorithms have been commonly and successfully used to estimate the relevance of the features to the target concept. These algorithms contain Relief, ReliefF and RReliefF. The original Relief algorithm can only be used to deal with classification problems with two classes. By contrast, the ReliefF algorithm used in this study is more robust and can deal with multiclass problems. Its detail process can be found in [36]. For the ReliefF algorithm, weight is the index of quality estimation for all attributes, and its value depends on the ability of attributes to distinguish between instances that are near to each other. The algorithm searches both for k of its nearest neighbors from the same class and each of the different classes when selecting an instance. They are called k nearest hits and nearest misses, respectively. Weights of all attributes are determined by their values for the instance, hits and misses. This is an iterative process. Selection of k hits and misses is the key to ensure greater robustness of the ReliefF algorithm in the process.

### 2.6. The Identification Algorithm of Rice ShB in Leaves

See5.0 Classifier was used for identifying rice leaves infected with ShB. The idea of this algorithm is the entropy criterion that the growth of the classification tree depends on the variable with the highest entropy or amount of information [37,38]. The entropy formula is as follows.

(2) $$\mathrm{Entropy}=-\overset{k}{\underset{j=1}{\sum }}\frac{n_{j}}{N}\times {\mathrm{Log}}_{2}\left(\frac{n_{j}}{N}\right)$$

where N is the total number of observations, k is the number of classes in N, and n_j_ is the number of observations belonging to the same class.

See5.0 Classifier has many advantages, including rulesets, boosting, evaluation systems and pruning options. These options are helpful to users to make an effective informative pattern. Classifiers were generated from the second data set and evaluated by 10-fold cross-validation in the study. Evaluation indexes included the average error rate and the standard errors of the means. The average error rate is the ratio of the total number of errors on the hold-out cases to the total number of cases. The standard errors of the means are an estimate of the variability of the results.

## 3. Results

### 3.1. Spectral Response of Leaf Blade Fractions with ShB Symptoms

**Figure 2:**
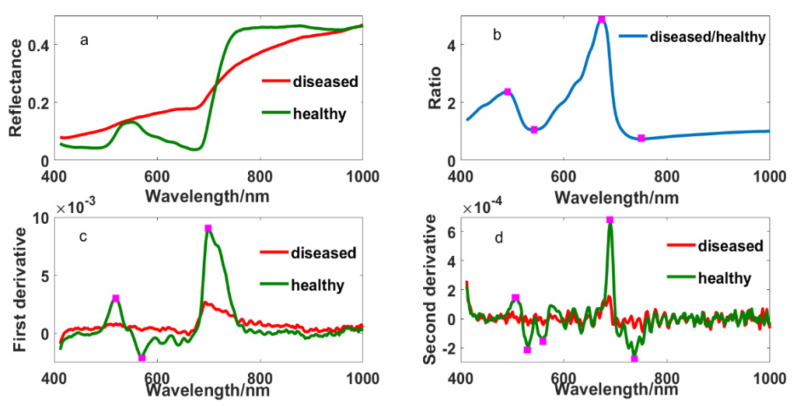
The spectrums of healthy and diseased leaf blade fractions: (**a**) original reflectance; (**b**) the ratio spectrum of diseased and healthy reflectance; (**c**) first derivative and (**d**) second derivative.

Figure 2 showed the original and different transformed spectral curves of healthy and diseased tissue fractions. Their differences were obvious in the original spectrum, which were mainly reflected in the blue edge, green peak, yellow edge, red edge and near-infrared band. Compared with the spectrum of healthy leaves, the spectrum of diseased leaves showed no absorption valleys in the blue edge and no peak in the green band but higher reflectance in the yellow edge. Furthermore, there was a weaker absorption valley, higher reflectance and a smaller “steep slope” in the red edge, and lower reflectance in the near-infrared band. Meanwhile, some specific wavelengths were found at 490, 508, 530, 542, 560, 568, 573, 638, 671, 700, 736 and 749 nm from ratio and derivative spectrums. This provides us with the possibility of selecting appropriate wavelengths to detect the disease. 

### 3.2. Construction of the Spectral Feature Library with Disease Symptom Proportions

#### 3.2.1. Statistical Analysis of “Three-Edge” Parameters

**Figure 3:**
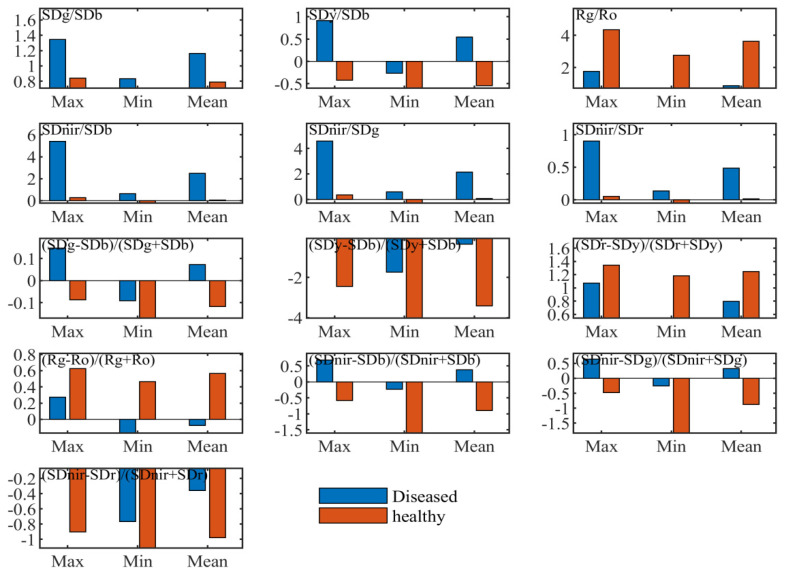
Descriptive statistics of some ratio and normalized indices with spectral amplitude and area parameters.

“Three-edge” parameters can quantitatively express spectral variations of diseased leaves in the blue edge, green peak, red valley, red edge and near-infrared band. Table 1 and Figure 3 illustrated statistical differences of the position, amplitude, area, ratio indices and normalized indices of “three parameters” between healthy and diseased leaf blade fractions, respectively. 

As shown in Table 1, variation coefficients of diseased leaf blade fractions were greater than those of healthy leaf blade fractions in various parameters of amplitude and area, except for yellow edge amplitude (Dy), yellow edge area (SDy) and near-infrared area (SDnir). The highest was red edge amplitude (Dr), which reached 0.365, followed by red edge area (SDr), red valley amplitude (Ro), blue edge amplitude (Db), blue edge area (SDb), green peak amplitude (Rg), and green peak area (SDg). This resulted from the different levels of ShB severity. Nevertheless, according to the maximum and minimum values of each parameter, it was clear that the whole ranges of Db, SDb, Dr, Ro, SDy, and SDnir were completely non-overlapping between ShB-infected and healthy leaf blade fractions, while these parameters of Rg, SDg, and SDr showed an inclusive or overlapping relationship. This finding suggests that the parameters of Db, SDb, Dr, Ro, SDy, SDnir facilitate the recognition of rice ShB. The positions of blue edge, green peak, yellow edge, red valley and red edge can move with changes in the biophysical and biochemical traits of vegetation. Spectral positions of healthy leaves were relatively stable in these waveband regions, which were located at 520, 551, 702 and 672 nm, respectively. However, spectral positions of leaf lesions varied widely due to different severity levels of rice ShB in the ranges of the blue edge, green peak, yellow edge, red valley and red edge. The blue edge position (BEP) changed from 501 to 530 nm centered around 520 nm. BEP had a shift towards shorter wavelengths when the disease was mild in severity. On the contrary, BEP moved to long wavelengths higher than 520 nm. The green peak position (GPP) was relatively stable between 553 and 560 nm centered around 560 nm. The yellow edge position (YEP) moved to longer wavelengths from 550 nm to 583 nm. The red valley position (RTP) was from 641 to 677 nm, mainly concentrated on 641 nm, which had a trend towards shorter wavelengths. The red edge position (REP) was mainly located at 692 and 695 nm. Compared to REP of healthy leaves, it was shown that REP shifted towards blue light.

Basic statistics of the ratio and normalized indices formed by the combination of “three-edge” areas and amplitudes were computed and analyzed. It was seen from the maximum and minimum values of every variable that the value ranges of some indices from diseased leaf blade fractions were different to those of healthy leaves. They are shown in Figure 3, including ratio indices of SDg/SDb, SDy/SDb, Rg/Ro, Sdnir/SDb, Sdnir/SDr, Sdnir/SDg and normalized indices of (SDg – SDb)/(SDg + SDb), (Sdy – SDb)/(Sdy + SDb), (SDr – Sdy)/(SDr + Sdy), (Rg – Ro)/(Rg + Ro), (Sdnir – SDb)/(Sdnir + SDb), (Sdnir – SDr)/(Sdnir + SDr), and (SDnir – SDg)/ (Sdnir + SDg). These variables indicate the ability of distinguishing the diseased leaves from the healthy ones. They contain not only the above individual parameters with the entirely non-overlapping value range but also the parameters with a range of full overlap and partial overlap. This implies that algebraic combination of spectral amplitudes and areas can amplify the amount of information and make use of parameters with weak disease information.

#### 3.2.2. Statistical Analysis of Narrow-Band Vegetation Indices

**Figure 4:**
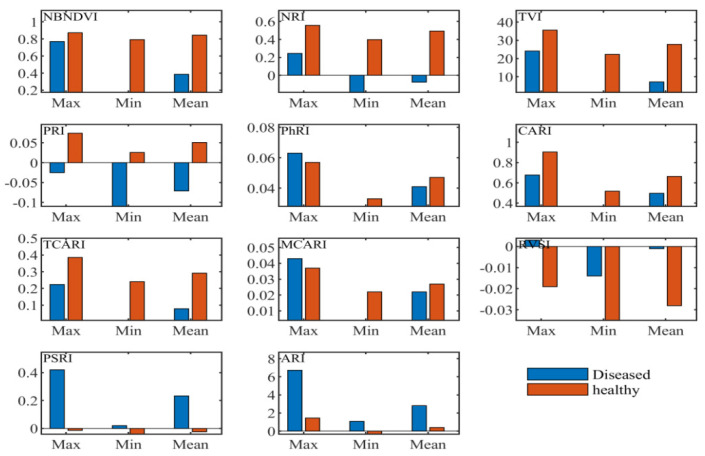
Descriptive statistics of narrow-band vegetation indices.

Narrow-band indices employed in this research included the common narrow-band ones such as the sensitivity to anthocyanin content, the indices independent of pigments, the chlorophyll absorption ratio index, the index related to photosynthetic efficiency, the and nitrogen reflectance index. According to the maximum, minimum, and coefficient of variation values shown in Figure 4, the vegetation indices, the narrow-band normalized difference vegetation index (NBNDVI), the nitrogen reflectance index (NRI), photochemical/physiological reflectance index (PRI), the transformed chlorophyll absorption and reflectance index (TCARI), the red-edge vegetation stress index (RVSI), and the plant senescence reflectance index (PSRI), can basically separate the healthy tissue from the diseased tissue. Throughout the structure of these vegetation indices, it was found that the wavelengths used in these variables include 500, 531, 550, 570, 670, 680, 700, 712, 732, 750, 752 nm, etc. These bands are consistent with the above sensitive characteristic wavelengths presented in Figure 2.

### 3.3. The Relevance of Each Variable in the Spectral Library to Rice ShB in Diseased Leaf Blade Fractions

**Figure 5:**
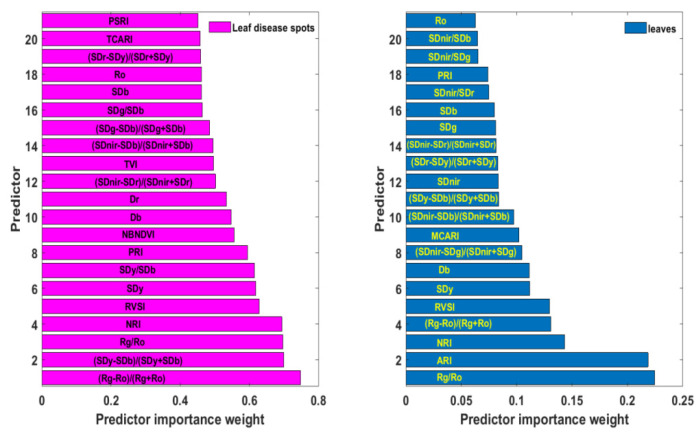
The importance order of spectral variables with the top 50% weight at leaf lesion and leaf scale.

The above spectral responses of rice ShB and statistical results of “three-edge” parameters and 11 narrow-band vegetation indices suggest that these variables can be considered for the spectral feature library of rice ShB. However, these parameters may contain specific spectral information of the disease or duplicate information, or both. The Relieff algorithm can link the predictor and the dependent variable to determine the importance of each predictor to the dependent variable. This importance is measured by the size of the weight. The higher the weight, the stronger the ability of this parameter to identify the disease. Figure 5 illustrated parameters with weights in the top 50% arranged in a descending order. Weights of (Rg − Ro)/(Rg + Ro), (Sdy − SDb)/(Sdy + SDb), Rg/Ro, NRI, RVSI, Sdy, Sdy/SDb, PRI, NBNDVI, blue edge amplitude (Db), red edge amplitude (Dr), (SDnir − SDr)/(SDnir + SDr) and triangular vegetation index (TVI) were close to or exceeded 0.5, and among these, the vegetation index (Rg − Ro)/(Rg + Ro) had the highest weight of 0.747. Except for blue edge amplitude and red edge amplitude, other predictors are all mathematical formulas combined by two or more single spectral features. This implies that indices formed by multiple single spectral features can expand disease information, eliminate noise and help to improve the ability to recognize rice ShB. In addition, these parameters corresponded to those with different range of values between healthy and diseased leaf blade fractions.

### 3.4. Capability of Detecting Rice ShB at the Leaf Scale by the Spectral Feature Library Constructed by Disease Symptom Fractions

Rice leaf lesions are difficult to extract due to the mutual shielding between the leaves, while leave samples are available from high spatial resolution images. Therefore, the examination of the application of spectral variables based on leaf lesions was given as follows at the leaf scale. Figure 5 presents the selected spectral parameters with top 50% weights by the ReliefF algorithm based on hyperspectral data of rice leaves. Compared with weights of various indicators at the lesion scale, the weight value of each feature at the leaf scale was significantly lower, with the maximum weight value being only 0.224. Furthermore, the characteristics with the top 50% weights were also different. The feature with the highest weight was Rg/Ro, not (Rg − Ro)/(Rg + Ro), which ranked fourth. Yellow edge area (SDy) and RVSI were both very important in the two kinds of data, ranking fifth and sixth. Other variables selected were blue edge amplitude (Db), blue edge area (SDb), red valley amplitude (Ro), NRI, PRI, (SDy − SDb)/(Sdy + SDb), (Sdnir − SDr)/(Sdnir + SDr), (Sdnir − SDb)/(Sdnir + SDb), and (SDr − SDy)/(SDr + SDy). It can be seen that the information of healthy proportions in the whole leaf will weaken and average out disease information, resulting in the importance differences of attributes. 

The discrimination model of rice ShB in the leaves was established to validate the reliability of features by the decision tree algorithm. The model took Rg/Ro with the highest weight as the predictive variable and used 10-fold cross validation. The average error rate of the model was 4.5%, and the standard error of the means was 3.0%. This demonstrates that the spectral library of “three-edge” parameters and narrow-band vegetation indices has certain recognition significance for rice ShB at the leaf or canopy scale. 

## 4. Discussion

### 4.1. Spectral Response Difference between Leaf Blade Lesions and Leaf Sheath Lesions

**Figure 6:**
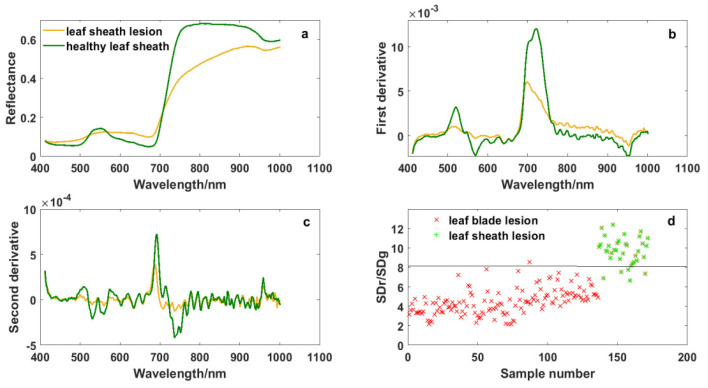
Spectral curves of leaf sheath lesions and the variable SDr/SDg: (**a**) Original spectrum, (**b**) first derivative transformation spectrum, (**c**) second derivative transformation spectrum, and (**d**) the ability of SDr/SDg to distinguish the leaf sheath lesions from the leaf blade lesions.

The damage caused by ShB is first on leaf sheaths, and then on leaf blades when ShB occurs in rice. Figure 2 and Figure 6 show the differences in spectral curves between the leaf blade lesions and the leaf sheath lesions. The absorption valley, reflectance peak and reflecting steep slope in the regions of blue edge, green light and red edge were more obvious in the leaf sheath lesions, though spectral curves of the leaf blade lesions and the leaf sheath lesions were similar in shape. These spectral curves are consistent with canopy spectral curves of rice infected with ShB in the report by Zhang et al. [18]. Meanwhile, it was observed from derivative spectral curves that the spectral response of leaf sheath lesions is different from the response of the leaf blade lesions in the red band of 670–750 nm and the near-infrared band of 915–980 nm. It is seen that rice infected with ShB, especially lesions, have a high impact on spectrum variations, which reflects the variation of absorption and reflection characteristics with wavelength.

“Three-edge” parameters and narrow-band vegetation indices were also extracted from the data of leaf sheath lesions, and the quality of these variables was estimated by statistical analysis and the ReliefF algorithm. Figure 6 shows that the variable SDr/SDg had the power to distinguish the leaf sheath lesions from the leaf blade lesions. The average of SDr/SDg in the leaf sheath lesions was 9.665, which was higher than that of the leaf blade lesions, and it could separate them well when the threshold value of SDr/SDg was 8. The feature SDr/SDg was the ratio of the red edge area and the green peak area, which enhanced the spectral differences between the leaf sheath lesions and the leaf blade lesions. Of course, with the change in rice variety and plant growth stage, the most significant characteristic may change as well, but it will be all among the “three-edge” parameters and related vegetation indices corresponding to the spectral response mechanism of the leaf sheath lesions and the leaf blade lesions.

### 4.2. The Physiological Basis of Spectral Characteristic Parameters and Vegetation Indices for Identifying Rice ShB

Rice plants develop different symptoms after being infected with the ShB fungus [3]. These symptoms on the leaves begin with small dark green patches, then manifest in the development of lesions and color changes into brown and gray, finally causing dead and dry leaves. It is worth noting that the different symptoms of rice ShB can be observed simultaneously in various portions of the leaf. Therefore, reflectance spectra will vary with the development stage of the lesions. Thus, “three-edge” parameters and narrow-band indices extracted from the hyperspectral data of leaves with different symptoms can capture spectral information of rice ShB showing different symptoms. Spectral information related to these symptoms results from the changes in physiological and biochemical activities in infected leaves, such as chlorophyll, carotenoid, anthocyanin, nitrogen, carbohydrates and protein [5,39]. Therefore, the various spectral characteristic parameters for identifying rice ShB on the leaves correspond to the physiological and chemical parameters inside the leaves. Spectral variables selected by the ReliefF algorithm were in the blue edge, green peak, yellow edge, red edge and near-infrared band, including blue edge amplitude (Db), yellow edge area (SDy), Rg/Ro, (Rg − Ro)/(Rg + Ro), and (SDnir − SDg)/(SDnir + SDg). Shifts in these positions to longer or shorter wavelengths have been used as indicators of vegetation stress [23]. The phenomenon of red-edge shift towards blue light can be mainly attributed to the changes in chlorophyll concentration of plants under the disease stress. These results are similar to those reported in previous studies [17,18,19]. High reflectance in the near-infrared band of 783–890 nm results from the multiple scattering in the inner tissue of leaves [40,41]. Compared with broad bands, narrow bands can provide additional information for better quantifying of the biophysical characteristics of agricultural crops. Hence, narrow-band vegetation indices can highlight the specific information of plants under stress to a large extent. Because rice ShB is caused by a fungal pathogen, some of the secretions from the fungus can also affect the normal metabolism of the host plant, causing a series of abnormal physiological and biochemical changes in the host plant. These include the changes in cell membrane permeability and electrolyte leakage, followed by changes in respiration and photosynthesis; in metabolism of nucleic acids, proteins, phenols and related enzymes; and in water physiology and others [3]. The anthocyanin reflectance index (ARI) reflects the change in anthocyanin content in leaves, and the red-edge vegetation stress index RVSI illustrates the variation of pigments in leaves. Rice ShB can also influence the photosynthetic efficiency of leaves [3]. The photochemical/physiological reflectance index (PRI) is related to photosynthetic efficiency. Besides, it has been reported that there are effects of different nitrogen, phosphorus, and potassium levels on the incidence of rice ShB [42]. The NRI vegetation index is also selected and has higher relevance to rice ShB at the leaf scale. However, further investigations are needed to verify these results by hyperspectral images obtained in the field due to many factors affecting extraction of rice leaves under the natural environments. 

## 5. Conclusions

ShB is a common disease in rice, and the levels of ShB severity are correlated with grain yield loss. The symptoms of the disease are characterized by water-soaked, circular to oblong, ellipsoid to ovoid or even irregularly elongated discolored lesions on the leaf sheaths and the leaf blades. This study used hyperspectral remote sensing technology to evaluate spectral response curves of leaf blade lesions to find specific characteristics for establishing the spectral feature library of rice ShB. We hope to apply these features to hyperspectral data at the leaf scale to explore the possibility of remote sensing monitoring of rice ShB at the regional or an even larger area scale. The spectrum curves of diseased leaves with lesions were significantly different from those of healthy leaves in original reflectance and derivative-transformed spectrum curves, especially in the blue edge, green peak, red valley, and red edge. “Three-edge” parameters and some narrow-band indices were selected and calculated based on spectral responses of the disease to construct the spectral feature library of rice ShB. These features closely linked to diseased leaf blade fractions with different development stages of symptoms by statistical analysis and the Relieff algorithm, including (Rg − Ro)/(Rg + Ro), (SDy − SDb)/(SDy + SDb), Rg/Ro, NRI, RVSI, yellow edge area, SDy/SDb, PRI, NBNDVI, and blue edge amplitude. Then, the spectral feature library was examined in the disease data set at the leaf scale. The model with Rg/Ro had higher classification accuracy. Rice ShB is different from other diseases of rice in that it initially occurs in leaf sheaths. Therefore, this study also discussed spectral response differences between the leaf sheath lesions and the leaf blade lesions. The spectral curve shapes between them were similar, but the leaf sheath lesions had more obvious characteristics of absorption valley, reflection peak and reflecting steep slope in the blue edge, green peak and red edge than the leaf blade lesions. The most significant feature was SDr/SDg. Although they were based on analysis of hyperspectral images acquired under laboratory conditions, these results can provide the basis for the development of portable diagnostic equipment for rice ShB based on multispectral wavebands.

## Figures and Tables

**Table 1:** Descriptive statistics of spectral position, amplitude and area.

Variables	Maximum		Minimum		Mean		C.V.	
	Diseased	Healthy	Diseased	Healthy	Diseased	Healthy	Diseased	Healthy
**Db**	0.002	0.004	0.001	0.003	0.001	0.003	0.222	0.105
**BEP**	530	523	501	515	517	520	0.017	0.002
**Rg**	0.209	0.173	0.076	0.111	0.148	0.132	0.196	0.106
**GPP**	560	555	553	548	560	551	0.002	0.003
**Dy**	0.001	0.000	0.000	0.000	0.001	0.000	0.221	1.527
**YEP**	583	555	550	550	558	551	0.016	0.001
**Dr**	0.008	0.011	0.001	0.008	0.003	0.009	0.365	0.080
**REP**	734	723	690	698	699	702	0.012	0.008
**Ro**	0.267	0.057	0.067	0.029	0.174	0.037	0.248	0.140
**RTP**	677	677	641	669	654	672	0.020	0.003
**SDb**	0.020	0.043	0.007	0.026	0.012	0.032	0.198	0.119
**SDg**	0.020	0.032	0.009	0.019	0.013	0.025	0.155	0.103
**SDy**	0.012	−0.014	−0.005	−0.022	0.006	−0.017	0.481	−0.087
**SDr**	0.147	0.207	0.030	0.124	0.058	0.157	0.282	0.136
**SDnir**	0.048	0.007	0.008	−0.007	0.027	0.002	0.287	1.102

C.V.: Coefficient of variation; Db: Blue edge amplitude; BEP: Blue edge position; Rg: Green peak amplitude; GPP: Green peak position; Dy: Yellow edge amplitude; YEP: Yellow edge position; Dr: Red edge amplitude; REP: Red edge position; Ro: Red valley amplitude; RTP: Red valley position; SDb: Blue edge area; SDg: Green peak area; SDy: Yellow edge area; SDr: Red edge area; SDnir: Near-infrared area.

## Appendix A

**Table A1:** Types of “three-edge” parameters.

Variables	Definition and Algorithm	References
Blue edge amplitude (Db)	Maximum value of the first derivative spectrum in the blue light band of 490 to 530 nm	Gong et al. (2002)
Blue edge position (BEP)	Waveband position corresponding to Db (nm)	Gong et al. (2002)
Green peak amplitude (Rg)	Maximum reflectance in the 510–560 nm green band	Gong et al. (2002)
Green peak position (GPP)	Waveband position corresponding to Rg (nm)	Gong et al. (2002)
Yellow edge amplitude (Dy)	Maximum value of the first derivative spectrum in the yellow light band of 550 to 582 nm	Gong et al. (2002)
Yellow edge position (YEP)	Waveband position corresponding to Dy (nm)	Gong et al. (2002)
Red valley amplitude (Ro)	Minimum reflectance in the 640–680 nm red band	Gong et al. (2002)
Red valley position (RTP)	Waveband position corresponding to Ro (nm)	Gong et al. (2002)
Red edge amplitude (Dr)	Maximum value of the first derivative spectrum in the red light band of 670 to 750 nm	Gong et al. (2002)
Red edge position (REP)	Waveband position corresponding to Dr (nm)	Gong et al. (2002)
Blue edge area (SDb)	sum of the first order derivative values within the blue edge	Gong et al. (2002)
Green peak area (SDg)	sum of the first order derivative values within the green band	Gong et al. (2002)
Yellow edge area (SDy)	sum of the first order derivative values within the yellow edge	Gong et al. (2002)
Red edge area (SDr)	sum of the first order derivative values within the red edge	Gong et al. (2002)
Near-infrared area (SDnir)	sum of the first order derivative values within the near-infrared rand of 783–890 nm	Gong et al. (2002)
SDg/SDb	Ratio of green peak to blue edge area	Zhang et al. (2015)
SDy/SDb	Ratio of yellow edge to blue edge area	Zhang et al. (2015)
SDr/SDb	Ratio of red edge to blue edge area	Horler et al. (1983)
SDr/SDg	Ratio of red edge to green peak area	Zhang et al. (2015)
SDr/SDy	Ratio of red edge to yellow edge area	Horler et al. (1983)
Rg/Ro	Ratio of green peak reflectance to red valley reflectance	Horler et al. (1983)
SDnir/SDb	Ratio of near infrared to blue edge area	Zhang et al. (2015)
SDnir/SDg	Ratio of near infrared to green peak area	Zhang et al. (2015)
SDnir/SDy	Ratio of near infrared to yellow edge area	Zhang et al. (2015)
SDnir/SDr	Ratio of near infrared to red edge area	Zhang et al. (2015)
Dr/SDr	Ratio of red edge amplitude to red edge area	Zhang et al. (2015)
(SDg − SDb)/(SDg + SDb)	The normalized difference of green peak and blue edge areas	Zhang et al. (2015)
(SDy − SDb)/(SDy + SDb)	The normalized difference of yellow edge and blue edge areas	Zhang et al. (2015)
(SDr − SDb)/(SDr + SDb)	The normalized difference of red edge and blue edge areas	Horler et al. (1983)
(SDr − SDg)/(SDr + SDg)	The normalized difference of red edge and green peak areas	Zhang et al. (2015)
(SDr − SDy)/(SDr + SDy)	The normalized difference of red edge and yellow edge areas	Horler et al. (1983)
(Rg − Ro)/(Rg + Ro)	The normalized difference of green peak reflectance and red valley reflectance	Horler et al. (1983)
(SDnir − SDb)/(SDnir + SDb)	The normalized difference of near infrared and blue edge areas	Zhang et al. (2015)
(SDnir − SDg)/(SDnir + SDg)	The normalized difference of near infrared and green peak areas	Zhang et al. (2015)
(SDnir − SDy)/(SDnir + SDy)	The normalized difference of near infrared and yellow edge areas	Zhang et al. (2015)
(SDnir − SDr)/(SDnir + SDr)	The normalized difference of near infrared and red edge areas	Zhang et al. (2015)

## Appendix B

**Table A2:** Hyperspectral vegetation indices for plant disease detection.

Indexes	Definition	Description or Formula	Literature
**NBNDVI**	Narrow-band normalized difference vegetation index	(R850 − R680)/(R850 + R680)	Thenkabail et al. (2000)
**NRI**	Nitrogen reflectance index	(R570 − R670)/(R570 + R670)	Filella et al. (1995)
**TVI**	Triangular vegetation index	0.5[120(R750−R550) − 200(R670 − R550)]	Broge and Leblanc (2001)
**PRI**	Photochemical/physiological reflectance index	(R531 − R570)/(R531 + R570)	Gamon et al. (1992)
**PhRI**	The physiological reflectance index	(R550 − R531)/(R550 + R531)	Gamon et al. (1992)
**CARI**	Chlorophyll absorption ratio index	(|(a670 + R670 + b)|/(a2 + 1)1/2) − (R700/R670), a = (R700 − R550)/150, b = R550 − (a − 550)	Kim et al. (1994)
**TCARI**	The transformed chlorophyll absorption and reflectance index	3[(R700 − R670) − 0.2(R700 − R550)(R700/R670)]	Haboudane et al. (2004)
**MCARI**	Modified chlorophyll absorption ratio index	[(R701 − R671) − 0.2(R701 − R549)]/(R701/R671)	Daughtry et al. (2000)
**RVSI**	Red-edge vegetation stress index	[(R712 + R752)/2] − R732	Merton and Huntington (1999)
**PSRI**	Plant senescence reflectance index	(R680 − R500)/R750	Merzlyak et al. (1999)
**ARI**	Anthocyanin reflectance index	(R550)^−1^ − (R700)^−1^	Gitelson et al. (2001)
